# Exploring the inter-factor network of psychological abuse, life-event stress, and suicidal behavior: evidence from Chinese college students

**DOI:** 10.3389/fpsyg.2026.1751556

**Published:** 2026-02-24

**Authors:** Jiawei Cao, Zhiming Huo, Xinchun Liu, Nan Meng, Tingting Tan

**Affiliations:** 1 Graduate School of Human Development and Environment, Kobe University, Kobe, Japan; 2 Key Research Base of Humanities and Social Sciences of the Ministry of Education, Academy of Psychology and Behavior, Tianjin Normal University, Tianjin, China; 3 Faculty of Psychology, Tianjin Normal University, Tianjin, China; 4 Tianjin Labor Economics School (The Second Advanced Technician School), Tianjin, China; 5 Mental Health Education Center, Tianjin University, Tianjin, China

**Keywords:** college students, life-event stress, network analysis, psychological abuse, suicidal behavior

## Abstract

**Background:**

Suicide remains a leading cause of death among young adults, yet traditional mediation models often oversimplify the complex and interactive processes linking childhood psychological abuse, stress, and suicidal behavior.

**Objective:**

This study applied a network analytical framework to examine the inter-factor associations among dimensions of childhood psychological abuse, life-event stress responses, and suicidal behavior in Chinese college students.

**Methods:**

A total of 693 first-year college students completed validated self-report measures of childhood psychological abuse, stress responses to negative life experiences, and suicidal behavior. Gaussian graphical models were estimated using EBICglasso, and bridge centrality indices were computed to identify key nodes linking abuse, stress, and suicidality. Network comparison tests were conducted to examine gender differences in network structure and global connectivity.

**Results:**

The estimated network showed dense within- and cross-domain connections among psychological abuse, stress responses, and suicidal behavior. Neglect, intrusion, and arousal emerged as prominent bridge nodes linking early psychological abuse and suicidal outcomes through stress-related pathways. Network structure was invariant across genders; however, males exhibited significantly higher global network strength than females (*p* < 0.05), indicating stronger overall connectivity among network components.

**Conclusion:**

These findings suggest that suicide risk emerges from dynamic interactions among interrelated psychological systems rather than from linear causal pathways. By identifying bridge nodes—particularly arousal and intrusive stress responses—this study highlights potential leverage points for targeted, trauma-informed, and culturally sensitive suicide prevention efforts among college students.

## 1 Introduction

Suicide is a global mental health crisis and a leading cause of premature mortality among young people (Bilsen, 2018; World Health Organization, 2023). In China, suicide accounts for nearly half of all unnatural deaths among college students (Yang and Li, 2015). Although multiple risk factors—including psychopathology, maladaptive coping, and stress—have been identified (Wu et al., 2018), how these factors interact to jointly shape suicide risk remains insufficiently understood.

Childhood psychological abuse, encompassing behaviors such as intimidation, neglect, devaluation, interference, and overindulgence, represents one of the most pervasive yet often overlooked forms of maltreatment (Liu and Wang, 2014; Baker et al., 2021). Accumulating evidence suggests that such early adversities are associated with long-term impairments in emotion regulation, increased hopelessness, and elevated suicidal ideation and behavior (Zhang et al., 2021; Rosenfield et al., 2022; Zhou et al., 2023). Negative life experiences may further exacerbate these vulnerabilities by eliciting maladaptive stress responses, thereby acting as proximal contributors to suicidal risk (Park et al., 2015; Chang et al., 2017). However, these constructs are inherently multidimensional, and their interrelations are unlikely to be adequately captured by a single linear mediating pathway.

From a network perspective, mental health problems are conceptualized not as manifestations of latent variables but as complex systems in which symptoms and psychosocial dimensions directly interact and reinforce one another (Borsboom and Cramer, 2013; Borsboom, 2017). This framework has been increasingly adopted in psychopathology research, offering a way to model mutual dependencies among components rather than assuming unidirectional causal chains (McNally, 2016; Robinaugh et al., 2020; Beard et al., 2016; McNally et al., 2015; Spiller et al., 2017; Fried and Cramer, 2017). Methodologically, psychological networks can be estimated to quantify conditional associations among dimensions and to evaluate the accuracy and stability of these estimates (Epskamp et al., 2018). Importantly, network metrics such as bridge centrality enable the identification of nodes that connect theoretically distinct communities, providing clinically relevant hypotheses about leverage points that are not captured by a single indirect effect in mediation or structural equation models (Jones et al., 2021).

Within this framework, stress is conceptualized as individuals’ psychological responses to broadly defined negative life experiences rather than to a single discrete traumatic event. Stress responses such as intrusion, avoidance, and arousal may serve as critical mechanisms through which early psychological abuse becomes linked to suicidal thoughts and behaviors. Network analysis enables the examination of how specific dimensions of childhood psychological abuse (e.g., emotional neglect, interference) interact with stress responses and form bridges to suicidal risk. Moreover, demographic contexts, including gender and experiences of being left behind, may further shape the configuration and connectivity of these networks (Liu et al., 2024).

Building on this abuse–stress–suicide framework, the present study adopts an inter-factor network approach to: (1) construct a co-occurrence network encompassing subdimensions of childhood psychological abuse, stress responses to negative life experiences, and suicidal behaviors; (2) identify bridge nodes linking abuse-related factors and suicidal risk; and (3) compare network structures across key demographic groups using network comparison tests. By capturing the multidimensional and interactive nature of these risk factors, this approach extends traditional mediation models and highlights potential leverage points for early intervention among college students.

## 2 Methods

### 2.1 Participants

This study recruited first-year college students from two institutions located in a city in Northern China. A questionnaire-based survey was administered one month after student enrollment. All procedures involving human participants were approved by the Ethics Committee of Tianjin Normal University [Protocol #(2024120203)] on December 2, 2024. Informed consent was obtained from all participants prior to survey administration. A small proportion of participants were under the age of 18; for these participants, assent was obtained from the individual, and written consent was obtained from a legal guardian/next of kin, in accordance with ethics committee requirements.

### 2.2 Research instruments

The Child Psychological Abuse Scale, revised by Pan et al. (2010), is a validated instrument designed to assess psychological abuse across five dimensions: intimidation, neglect, denigration, interference, and indulgence. Developed within the context of Chinese sociocultural norms, the scale comprises three factors and eight items. It has demonstrated strong psychometric properties, affirming its applicability for research use (Pan et al., 2010).

The Impact of Event Scale-Revised (IES-R) was used to assess participants’ psychological stress responses to broadly defined negative life experiences. The IES-R consists of 22 items measuring three dimensions: intrusion, avoidance, and arousal. In the present study, participants were not instructed to anchor their responses to a single discrete traumatic event. Instead, they were asked to rate the extent to which they had experienced intrusive thoughts, avoidance behaviors, and physiological arousal in response to stressful or distressing life experiences in general. Accordingly, the IES-R was used as an index of general event-related stress responses rather than as a measure of post-traumatic stress disorder. All items were rated on a 5-point Likert scale ranging from 0 (“not at all”) to 4 (“extremely”), with higher scores indicating more severe stress responses (Guo et al., 2007). Throughout this manuscript, the term “life-event stress” refers specifically to stress response dimensions (intrusion, avoidance, and arousal) assessed by the IES-R, rather than to objective exposure to discrete life events.

The Suicidal Behavior Questionnaire, developed by Yang et al. (2021), is a screening tool designed to assess suicide risk among students. The questionnaire comprises 12 items covering multiple aspects of suicidal behavior, including suicidal ideation, prior suicide attempts, and recent suicide attempts. Previous studies have demonstrated that the scale has good internal consistency and satisfactory psychometric properties in student populations (Yang et al., 2021). In the present study, items were grouped according to their conceptual content to construct three network nodes: suicidal ideation, previous suicide attempts, and suicide attempts.

### 2.3 Survey methodology

A random sampling strategy was employed, and electronic questionnaires were distributed via online platforms. Data collection was conducted through group testing sessions organized by class and administered in a standardized format. All of the procedures were supervised by principal investigators with extensive experience in psychological research. A total of 853 questionnaires were returned. After excluding 160 questionnaires for incompleteness and straightlining/invalid patterns, 693 valid questionnaires were retained for analysis. The effective response rate was calculated as 693/853 = 81.243%. The final sample consisted of 175 male and 518 female participants. The mean age of participants was 20.26 years (SD = ±1.27 years).

### 2.4 Sample size estimation for network analysis

According to the methodological recommendations for psychological network modeling, the minimum sample size should exceed the total number of parameters estimated—that is, the number of nodes plus the number of possible edges—to ensure the stability and accuracy of parameters (Epskamp et al., 2018). In the present study, the network included five nodes representing the dimensions of childhood psychological abuse, three nodes representing suicidal behavior, and three nodes representing negative life-event impacts, resulting in a total of 11 nodes. The total number of possible edges among these nodes was 55. Therefore, the minimum required sample size for stable estimation was 66.

A total of 693 valid cases were collected in this study. This sample size satisfies the general heuristic that stability improves substantially when the number of observations is at least an order of magnitude greater than the number of model parameters (Epskamp et al., 2018). Moreover, prior simulation studies and tutorials have emphasized the importance of verifying network robustness through bootstrapping and case-dropping procedures, especially when estimating regularized partial correlation networks (Epskamp and Fried, 2018; Constantin et al., 2023). Consequently, the current sample provides sufficient power and reliability for network estimation and centrality analyses.

### 2.5 Network node construction

All network nodes were continuous subscale scores. For each instrument, item-level responses were aggregated into subscale totals following the original scoring manuals. Specifically, IES-R items were summed into intrusion, avoidance, and arousal subscales; CPMS items were summed into intimidation, neglect, denigration, interference, and indulgence subscales; and SBSQ items were aggregated into suicidal ideation, previous suicide attempts, and suicide attempts scores according to the instrument-specific scoring rules. The complete node-to-item mapping, response options, and scoring procedures are provided in Supplementary Table S1.

### 2.6 Statistical methods

The data were entered using Excel 2024, and descriptive statistics were computed using SPSS 27.0. Network analyses were performed in R (version 4.5.1).

#### 2.6.1 Network estimation

Each node represented a subscale/dimension score (continuous variables) from the corresponding instruments. Cases with missing values were removed during data cleaning, resulting in a complete dataset for network estimation. We estimated a regularized Gaussian graphical model (GGM) using the EBICglasso procedure (tuning parameter *γ* = 0.5), and edges represent regularized partial correlations (conditional associations) between nodes after controlling for all other nodes. Networks were estimated from Spearman correlation matrices (pairwise complete observations) and visualized using a spring layout.

#### 2.6.2 Predictability

Node predictability was quantified as the proportion of explained variance (*R*^2^) using the mgm package (Gaussian model; type = “g,” level = 1) with cross-validated regularization. Predictability was displayed as pie charts in the network figure.

#### 2.6.3 Communities and bridge centrality

Communities were defined *a priori* according to the abuse–stress–suicide framework: stress responses (avoidance, intrusion, arousal), psychological abuse (intimidation, neglect, denigration, interference, indulgence), and suicidal behavior (suicidal ideation, previous suicide attempts, suicide attempts). Bridge centrality indices (bridge strength and bridge expected influence) were computed to identify nodes that connect these communities.

#### 2.6.4 Accuracy and stability

To evaluate the robustness of the estimated network, we conducted nonparametric bootstrapping (1,000 samples) to derive 95% confidence intervals for edge weights. The stability of centrality indices was assessed using case-dropping bootstrapping (2,500 samples). We report the correlation stability (CS) coefficient, which reflects the maximum proportion of cases that can be dropped while retaining a correlation of 0.70 with the original centrality estimates in at least 95% of bootstrap samples. Values above 0.25 are considered acceptable and values above 0.50 indicate good stability.

#### 2.6.5 Network comparison test

We compared network structures across gender groups using the network comparison test (NCT) with 1,000 permutations (*γ* = 0.5; weighted networks; paired = FALSE). We tested (i) network structure invariance, (ii) global strength invariance, and (iii) edge-specific differences. To account for multiple testing in edge-wise comparisons, *p*-values were adjusted using the false discovery rate (FDR) procedure with the Benjamini–Hochberg method (*q* < 0.05).

## 3 Results

### 3.1 Preliminary analysis

The internal consistency coefficients for the total score and five dimensions of the Child Psychological Abuse Scale, namely intimidation, neglect, denigration, interference, and indulgence, were 0.956, 0.927, 0.880, 0.919, 0.842, and 0.670, respectively. For the Impact of Life Events Scale, the total and subscale internal consistency coefficients, namely intrusion, arousal, and avoidance, were 0.962, 0.906, 0.884, and 0.869, respectively. The internal consistency coefficient for the Suicidal Behavior Questionnaire was 0.908. These findings indicate that the instruments possess good overall reliability; however, the indulgence dimension of the Psychological Abuse Scale demonstrated relatively lower internal consistency, though it remained within the acceptable range. As presented in Table 1, the factor loadings for the indicators related to the latent variables, namely childhood psychological abuse and impact of life events, were predominantly high, with most values exceeding 0.7, indicating strong representativeness of the constructs. Although the factor loading for the indulgence dimension was comparatively lower, it exceeded 0.3 and was statistically significant; thus, it was retained in the analysis. The extracted average variance extracted values for both latent variables were above 0.5, and their composite reliability values surpassed the standard threshold of 0.6, confirming good convergent validity (see Table 2).

**Table 1:** Factor loadings of the main variables.

Dimension	Estimate	Average variance extracted	Composite reliability
Childhood psychological abuse		0.652	0.900
Intimidation	0.938		
Neglect	0.815		
Denigration	0.933		
Interference	0.850		
Indulgence	0.350		
Impact of life events		0.826	0.934
Avoidance	0.896		
Intrusion	0.956		
Arousal	0.872		
Suicidal behavior			

**Table 2:** Correlations among childhood psychological abuse, life-event stress, and suicidal behavior.

Dimension	Avoidance	Intrusion	Arousal	Intimidation	Neglect	Denigration	Interference	Indulgence	Suicidal ideation	Previous suicide attempts	Suicide attempts
Avoidance	1										
Intrusion	0.861^***^	1									
Arousal	0.770^***^	0.832^***^	1								
Intimidation	0.496^***^	0.516^***^	0.541^***^	1							
Neglect	0.449^***^	0.459^***^	0.496^***^	0.765^***^	1						
Denigration	0.426^***^	0.441^***^	0.511^***^	0.873^***^	0.764^***^	1					
Interference	0.422^***^	0.448^***^	0.485^***^	0.793^***^	0.672^***^	0.804^***^	1				
Indulgence	0.239^***^	0.281^***^	0.292^***^	0.326^***^	0.417^***^	0.319^***^	0.298^***^	1			
Suicidal ideation	0.377^***^	0.386^***^	0.388^***^	0.412^***^	0.335^***^	0.340^***^	0.359^***^	0.159^***^	1		
Previous suicide attempts	0.287^***^	0.305^***^	0.315^***^	0.416^***^	0.370^***^	0.408^***^	0.430^***^	0.166^***^	0.784^***^	1	
Suicide attempts	0.195^***^	0.198^***^	0.208^***^	0.304^***^	0.325^***^	0.351^***^	0.384^***^	0.159^***^	0.537^***^	0.815^***^	1
Total score of suicidal behavior	0.294^***^	0.305^***^	0.314^***^	0.405^***^	0.378^***^	0.407^***^	0.435^***^	0.178^***^	0.798^***^	0.965^***^	0.917^***^

^*^p < 0.05, ^**^p < 0.01, and ^***^p < 0.001.

To assess data normality, the skewness and kurtosis coefficients of continuous variables were examined and compared to a normal distribution. The suicidal behavior variable deviated significantly from normality due to its content characteristics. A logarithmic transformation was performed to address skewness; however, no significant differences were observed between the transformed and untransformed variables. Therefore, untransformed data were used in subsequent analyses for interpretability.

Harman’s single-factor test was conducted to evaluate common method bias. Eleven factors with eigenvalues greater than 1 were extracted, and the first factor accounted for 30.286% of the variance, which was well below the 40% threshold, indicating that serious common method bias was not present in the dataset.

### 3.2 Network analysis results

**Figure 1:**
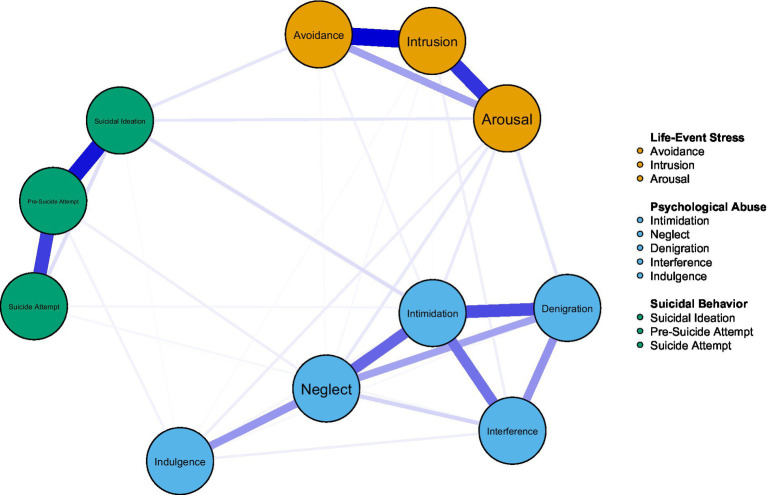
Co-occurrence network of psychological abuse, life-event stress, and suicidal behavior.

A psychological network model consisting of 11 nodes (including life-event stress, psychological abuse, and suicidal behavior variables) was estimated. The resulting network contained 35 nonzero edges out of 55 possible connections (63.6%), with a mean edge weight of 0.087. The estimated network structure is visualized in Figure 1. Strong connections were observed among the life-event stress nodes of avoidance, intrusion, and arousal. Within the psychological abuse domain, nodes such as neglect, intimidation, denigration, and interference were also densely interconnected. Additionally, the strongest edge was observed between suicidal ideation and suicide attempt, indicating a close internal linkage among suicidal behavior nodes.

**Figure 2:**
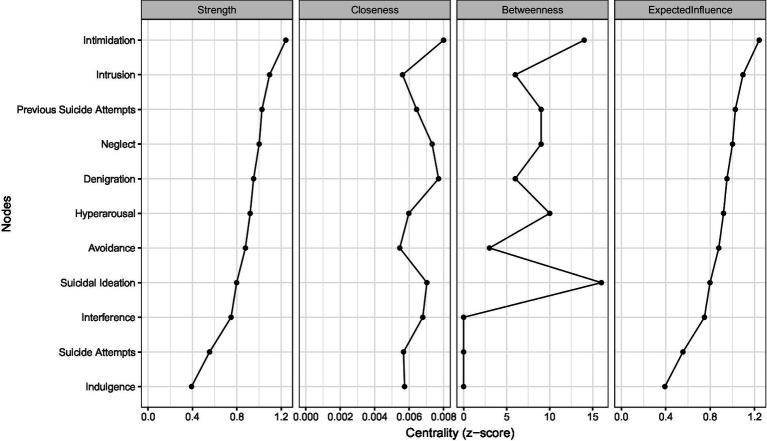
Centrality indices of nodes in the inter-factor network.

Centrality indices are presented in Figure 2. Overall, Intimidation exhibited the highest values for both strength and expected influence, suggesting that it plays a pivotal role within the network. Intrusion and neglect followed closely, indicating their substantial influence on other nodes. In contrast, indulgence and suicide attempt had lower centrality scores.

**Figure 3:**
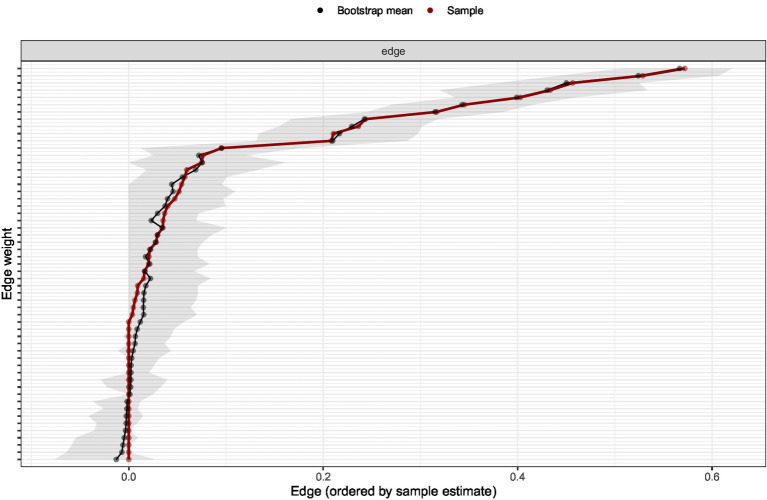
Bootstrapped confidence intervals of edge weights.

To assess the accuracy of edge weight estimation, a nonparametric bootstrap procedure was conducted. The bootstrap analysis revealed relatively narrow confidence intervals for most edge weights, indicating satisfactory estimation accuracy (Figure 3).

A network comparison test (NCT) was conducted to examine gender differences in network structure. The overall network structure did not differ significantly between males and females (M = 0.409, *p* = 0.081), suggesting a largely comparable edge-weight configuration across genders. However, global strength differed significantly (S = 1.328, *p* = 0.042), with higher overall connectivity in males than females (global strength = 6.17 vs. 4.84). Two edges showed nominal gender differences (avoidance–indulgence, *p* = 0.015; suicidal ideation–suicide attempts, *p* = 0.013); however, none remained significant after false discovery rate correction (Benjamini–Hochberg, *q* < 0.05).

### 3.3 Bridge analysis results of expected influence

**Figure 4:**
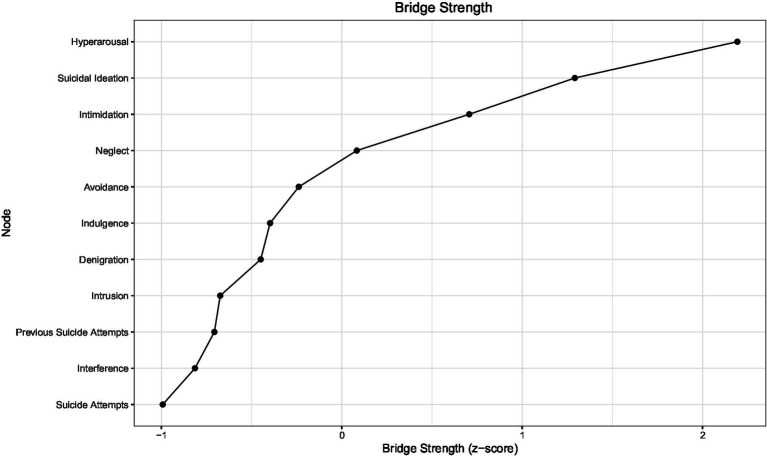
Standardized bridge strength (*Z*) of nodes connecting psychological abuse, stress, and suicidal behavior.

**Figure 5:**
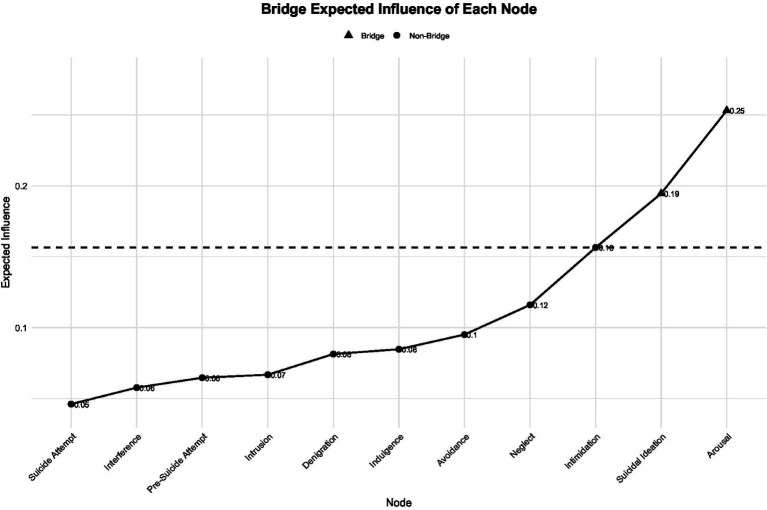
Bridge expected influence of nodes in the inter-factor network.

**Figure 6:**
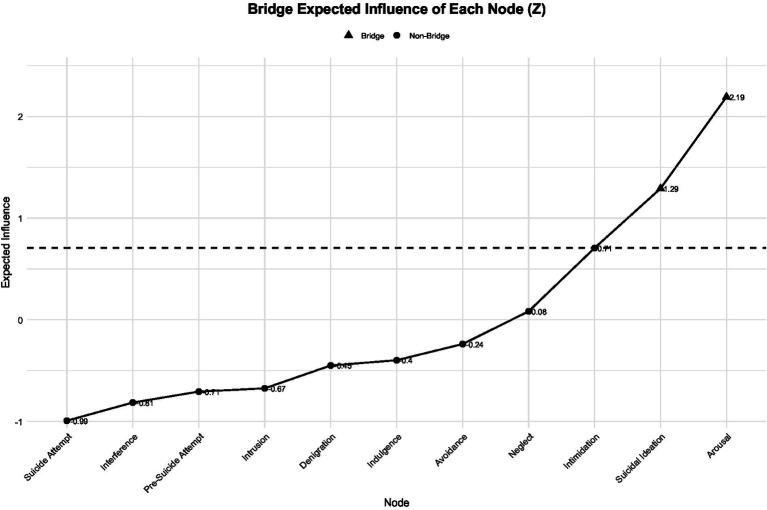
Standardized bridge expected influence (*Z*) of nodes.

The results of the bridge analysis are presented in Figures 4–6. In terms of standardized bridge strength, arousal, suicidal ideation, and intimidation exhibited the highest scores, indicating that these nodes serve as key connectors between distinct symptom communities—namely, life-event stress, psychological abuse, and suicidal behavior (Figure 4).

In terms of bridge expected influence (EI), arousal demonstrated the strongest bridge effect (EI = 0.25), followed by suicidal ideation (EI = 0.19) and intimidation (EI = 0.16), all exceeding the average bridge influence threshold (dashed line). Bridge expected influence values for all nodes are shown in Figure 5. The standardized bridge expected influence (*Z*) results further confirmed that arousal (*Z* = 2.19) and suicidal ideation (Z = 1.29) were the most influential bridge nodes within the network (Figure 6).

To evaluate the stability of the bridge expected influence metric, a case-dropping bootstrap procedure was conducted. The results indicated that the CS-coefficient for EI was 0.517, suggesting moderate to good stability across subsampling conditions. According to Epskamp et al. (2018), a CS value above 0.25 is considered acceptable, and values exceeding 0.5 indicate good stability; thus, the bridge expected influence in this study can be regarded as relatively robust. Furthermore, the overall stability pattern of the centrality indices was consistent, with both strength and expected influence reaching the highest tested stability level (CS = 0.75), whereas closeness (CS = 0.205) and betweenness (CS = 0.283) exhibited comparatively lower stability.

## 4 Discussion

### 4.1 Network structure of psychological abuse, life-event stress, and suicidal behavior

This study adopted a network analytical framework to explore the complex interrelations among childhood psychological abuse, life-event stress, and suicidal behavior in Chinese college students. The resulting network demonstrated dense within- and cross-construct connections, indicating that these domains are highly interdependent rather than linearly linked. This finding supports the conceptualization of psychopathology as a complex adaptive system in which symptoms or psychosocial dimensions mutually reinforce each other (Borsboom and Cramer, 2013; Bringmann et al., 2022).

Neglect and intrusion emerged as highly influential nodes closely involved in the bridging pathways linking early psychological abuse to suicidal behavior through stress-related mechanisms. Neglect—often manifesting as emotional deprivation or parental disengagement—has been shown to undermine emotional regulation and interpersonal trust, thereby heightening vulnerability to stress-related maladjustment and suicidality. Intrusive experiences, reflecting unwanted distressing thoughts or memories, have similarly been associated with trauma reactivation and suicidal ideation in previous research. The strong interconnections observed among stress-related nodes such as arousal, avoidance, and intrusion align with evidence that physiological arousal and cognitive intrusions sustain maladaptive feedback loops between stress and suicidality (Robinaugh et al., 2020).

Collectively, these results extend prior findings by suggesting that suicidal behavior risk emerges not from a single mediating process but from dynamic, self-reinforcing interactions among abuse, stress, and affective dysregulation. Such a perspective moves beyond traditional mediation models, providing a more ecologically valid representation of suicide risk formation.

### 4.2 Bridge centrality and potential intervention targets

The bridge centrality analysis identified arousal, intimidation, and suicidal ideation as nodes with the strongest bridging influence between the abuse–stress–suicide communities. Bridge nodes are theorized to play a crucial role in transmitting activation across different symptom clusters or constructs, acting as “gateways” through which one domain influences another (Robinaugh et al., 2020; Jones et al., 2021). In this study, arousal showed the highest bridge expected influence, suggesting that physiological and emotional hyperactivation may serve as a central conduit linking external stressors to internalized self-destructive tendencies.

This finding resonates with prior research indicating that arousal maintains suicidal ideation by amplifying negative affect and impulsive responses under stress. Targeting such bridge nodes may therefore represent an efficient strategy for prevention and intervention. Mindfulness-based stress reduction and trauma-focused cognitive behavioral interventions have been shown to reduce physiological arousal and intrusive thought activation, thereby disrupting maladaptive connectivity across domains (Bringmann et al., 2022). Enhancing self-compassion and emotion regulation may further buffer the transmission of stress effects between abuse-related and suicidal symptoms.

In practical terms, bridge centrality findings help identify specific leverage points within the psychological network that can be prioritized for early intervention. For example, programs emphasizing emotional awareness and self-regulatory training could attenuate the cascading influence of arousal and intimidation, thus reducing downstream suicidal risk.

### 4.3 Theoretical implications

By employing an inter-factor network approach, this study expands the psychological abuse–stress–suicide framework beyond traditional linear models. The observed network configuration empirically supports the notion that suicidal behavior arises from interactive, mutually reinforcing processes rather than isolated causal chains. This approach is in line with the network theory of mental disorders, which views psychopathological phenomena as emergent properties of systems composed of interconnected components (Borsboom and Cramer, 2013; Bringmann et al., 2022).

This study adopted a network analytical lens to delineate the complex inter-factor associations among childhood psychological abuse, negative life-event stress, and suicidal behavior in a college student sample. The results identified neglect, intrusion, and arousal as pivotal bridge nodes facilitating the propagation of network activation from early maltreatment through stress reactivity to suicidal outcomes. Network comparison analyses indicated that the overall network structure was invariant across genders; however, males exhibited significantly higher global network strength, suggesting stronger overall connectivity among network components.

### 4.4 Cultural/contextual interpretation

When interpreting the network structure and key nodes observed in this study, it is important to consider the unique sociocultural factors present in China. First, Chinese society has long emphasized academic achievement and familial expectations, placing immense pressure on students, not only from their own aspirations but also from parental and school demands, which significantly magnify this stress. Academic pressure thus becomes one of the central systemic factors influencing mental health (Jiang et al., 2021; Xu et al., 2024). In this environment, academic stress is more likely to enhance sensitivity to negative cognitions and physiological arousal, which may increase the interconnectivity between stress responses and mental health symptoms.

Secondly, mental health stigma remains pervasive in Chinese culture. This cultural tendency not only reduces the likelihood of individuals seeking help but also may cause nodes related to self-denial, avoidance, and emotional neglect to become more tightly linked within the core mental health network (Liu, 2024; Yang et al., 2019; Chen et al., 2025). The presence of stigma means individuals are more likely to internalize and suppress emotions, leading to increased psychological conflict and avoidance patterns. This could explain why nodes such as neglect, intrusion, and arousal emerge as critical bridge nodes in the network.

Furthermore, traditional Chinese family structures and parent–child interaction patterns emphasize authority, compliance, and family harmony. When communication within the family is insufficient or emotional neglect is present, individuals may lack effective emotional support when dealing with stress or failure experiences, which strengthens the connection between negative emotions and behavioral responses within the network (Yu et al., 2025). Changes in family functioning could therefore amplify the bridging function of nodes like neglect and intrusion in the network.

Thus, the results of this study can be interpreted through the lens of Chinese cultural factors: strong academic pressure, family structure and expectations, and societal stigma all contribute to the shaping of mental health network structures, with certain nodes playing a more critical bridging role. This highlights the importance of considering sociocultural factors in the activation and connectivity patterns of nodes when developing intervention strategies.

### 4.5 Mechanistic insights—PTSD intrusion-avoidance cycle and emotion regulation theory

When examining the mechanisms behind the key bridging nodes identified in this study (such as neglect/intrusion/arousal), we can deepen our understanding by drawing from the post-traumatic stress disorder (PTSD) symptom cycle and emotion regulation theory. PTSD research suggests that the core symptoms of this disorder revolve around a dynamic cycle between intrusive experiences, avoidance behaviors, and physiological arousal (the DSM-5 symptom framework includes intrusion, avoidance, cognitive/emotional changes, and arousal responses) (Weston, 2014). In this cycle, intrusive experiences (such as distressing recurring memories) often trigger the individual to attempt avoidance or suppression to reduce the painful feelings, but this avoidance strategy actually impedes emotional processing, leading to increased arousal and negative emotions, thus creating a difficult-to-break psychological feedback loop (Miethe et al., 2023).

From the perspective of emotion regulation theory, individuals who have experienced trauma often resort to maladaptive emotion regulation strategies (such as rumination, thought suppression, and experiential avoidance). While these strategies may provide short-term relief from emotional distress, they reinforce the maladaptive cycle of negative cognition and physiological arousal over time, preventing effective emotional regulation and balance (O’Brien et al., 2024). These maladaptive regulation strategies are likely to manifest as strong connections and bridging effects between nodes in the network. For example, highly intrusive thoughts or physiological arousal can “spread” to other psychological symptom nodes, maintaining the high connectivity and activity of the overall network.

When we combine this theoretical mechanism with the findings of this study, the high centrality of neglect, intrusion, and arousal nodes in the mental health network may reflect an emotional regulation dilemma faced by individuals when encountering stress or negative experiences. Intrusive cognitions trigger heightened arousal and avoidance responses, which not only make these nodes more active but also bridge different symptom communities. This aligns with the PTSD model of the intrusion-avoidance cycle: intrusion → avoidance → arousal → intrusion, which sustains the maladaptive feedback loop and promotes the interdependence of symptoms across various domains. By incorporating the PTSD mechanism and emotion regulation theory into the interpretation of the network’s bridging nodes, we can better understand why these nodes play a critical bridging role and why interventions should focus on addressing maladaptive emotion regulation strategies and the connectivity mechanisms between key nodes in the network.

### 4.6 Limitations and future directions

Several limitations should be noted. First, the cross-sectional design restricts causal inference and limits the capacity to examine temporal fluctuations in network dynamics. Longitudinal or experience-sampling studies are needed to capture the evolving structure of abuse–stress–suicide interactions over time (Bringmann et al., 2022). Second, reliance on self-report questionnaires introduces potential recall bias and common method variance. Future research incorporating behavioral, physiological, and ecological measures would yield more robust insights. Third, although the sample size was sufficient for stable estimation, participants were drawn from a single region in Northern China; thus, replication in diverse cultural and geographical contexts is warranted to ensure generalizability.

Despite these limitations, this study offers novel theoretical and methodological contributions by elucidating the inter-factor network structure linking psychological abuse, stress exposure, and suicidal behavior in Chinese college students. The findings reinforce the need to conceptualize suicide risk as a dynamic system of interconnected psychological processes.

## 5 Conclusion

This study adopted a network analytical lens to delineate the complex inter-factor associations among childhood psychological abuse, negative life-event stress, and suicidal behavior in a college student sample. The results identified neglect, intrusion, and arousal as pivotal bridge nodes facilitating the propagation of network activation from early maltreatment through stress reactivity to suicidal outcomes.

These insights highlight the importance of intervention strategies targeting bridge nodes—especially arousal and intrusive cognitions—to disrupt maladaptive network connectivity. Integrating emotional regulation training, trauma-informed therapy, and stress reduction practices may attenuate activation flow across the network. By reconceptualizing suicide risk as a dynamic system of interacting factors, this research advances both theoretical and practical understanding of how early adversity contributes to suicidality, offering culturally grounded implications for suicide prevention and mental health education in college settings.

## Data Availability

The datasets presented in this article are not readily available because due to the sensitive nature of the questions asked in this study, survey respondents were assured that all raw data would remain confidential and would not be shared. Requests to access the datasets should be directed to Tingting Tan, tantingting0218@163.com.
